# Boerhaave's syndrome: a differential diagnosis of chest and abdominal pain

**DOI:** 10.1590/0100-3984.2016.0138

**Published:** 2018

**Authors:** Thiago Almeida Ribeiro, Laura Torres da Costa Cordoval, Edgard de Magalhães Viana Neto, Marcelo Almeida Ribeiro, Emília Guerra Pinto Coelho Motta

**Affiliations:** 1 Hospital Mater Dei - Radiologia, Belo Horizonte, MG, Brazil

**Figure 1:**
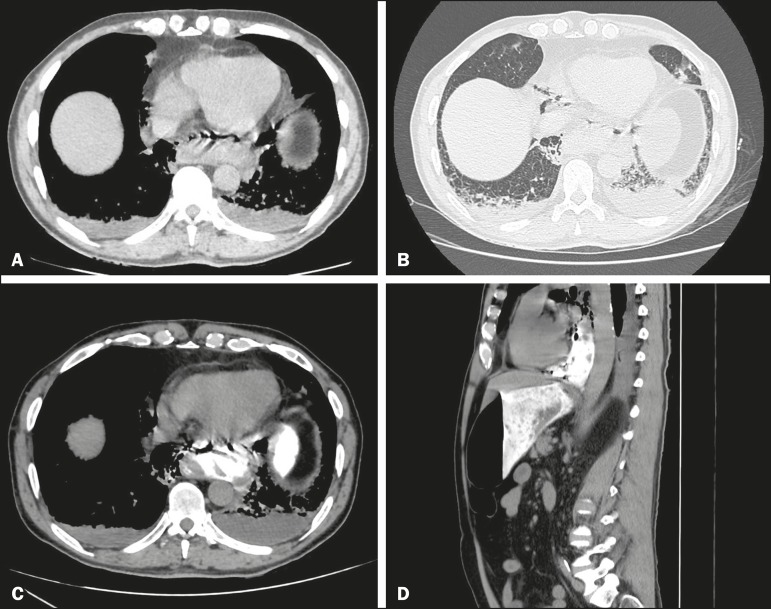
Axial CT of the thorax, with mediastinal and lung window settings (A and B, respectively), showing pneumomediastinum and a collection in the region of the distal thoracic esophagus. Oral contrast-enhanced CT scan of the abdomen, in the axial and sagittal planes (C and D, respectively), showing leakage of ingested material into the paraesophageal collection.

Dear Editor,

A 61-year-old male patient presented with a two-day history of diarrhea and vomiting, reporting dyspnea, as well as severe lower abdominal and thoracic pain with irradiation to the precordium and left shoulder. Physical examination showed a rigid abdomen and reduced breath sounds, with coarse crackles in both lung bases. Computed tomography (CT) of the thorax showed posterior pneumomediastinum and periesophageal densification by heterogeneous content (Figures 1A and 1B), and CT of the abdomen, complemented with a small amount of iodinated oral contrast, revealed extravasation to the posterior mediastinum (Figures 1C and 1D). In the emergency room, the patient had another episode of vomiting, which was followed by desaturation. He was immediately transferred to the operating room, where he underwent an extensive surgical procedure, during which esophageal perforation was identified in the distal third of the intrathoracic region. The perforation was closed, and the gastric contents were drained. The patient evolved to hemodynamic instability and was transferred to the intensive care unit.

Boerhaave's syndrome is the spontaneous perforation of the esophagus resulting from a sudden increase in intraesophageal pressure combined with negative intrathoracic pressure^(1)^. It is a rare condition, with an annual incidence of only 3.1 cases/ 1,000,000 population. Approximately 15% of esophageal perforations occur spontaneously, and the mortality rate exceeds 40%^(2,3)^.

Although Boerhaave's syndrome-related perforation occurs most commonly in the posterolateral intrathoracic aspect of the esophagus^(1)^, it can also occur in the cervical and intra-abdominal regions. The condition results in mediastinal contamination by gastric contents, precipitating chemical mediastinitis, with the possibility of evolution to bacterial infection and necrosis^(4)^.

Patients with Boerhaave's syndrome typically develop signs and symptoms of severe chest pain and subcutaneous emphysema. However, one third of such patients develop atypical symptoms or are admitted to the hospital with respiratory failure or shock^(4,5)^. Patients with cervical perforations can present with local pain, dysphagia, and dysphonia, as well as tension on sternocleidomastoid muscle palpation and crackles due to subcutaneous emphysema. In addition to Boerhaave's syndrome, the differential diagnoses of chest and abdominal pain should include myocardial infarction, pulmonary embolism, aortic dissection, and pancreatitis^(1)^.

Conventional radiography, barium swallow, and, especially, contrast-enhanced CT are of great value for the timely detection of Boerhaave's syndrome. CT shows the lungs, mediastinum, pleura, and aorta in greater detail, as well as having greater sensitivity in the detection of fluid collections. The findings corroborating rupture include esophageal edema with parietal thickening; perilesional fluid collections with or without a gaseous component; mediastinal widening; and fluid or air in the pleural and retroperitoneal spaces.

In cases of esophageal rupture, the basic therapeutic options include conservative treatment, endoscopic procedures, and surgery^(6,7)^. The conservative treatment consists in the interruption of oral food intake, together with fluid administration, enteral nutrition, antibiotic therapy, the use of beta-blockers, and drainage of the perilesional collections. Endoscopic therapy with stent placement can be reserved for cases in which there is early diagnosis, without contamination. Finally, the indication for surgical treatment, which varies from local debridement to the extensive resection of the esophagus, depends on factors such as the extent of the rupture, concomitant diseases, and the presence of contamination or signs of sepsis.
